# Phosphorylation and Activation of RhoA by ERK in Response to Epidermal Growth Factor Stimulation

**DOI:** 10.1371/journal.pone.0147103

**Published:** 2016-01-27

**Authors:** Junfeng Tong, Laiji Li, Barbara Ballermann, Zhixiang Wang

**Affiliations:** 1 Department of Medical Genetics, Faculty of Medicine and Dentistry, University of Alberta, Edmonton, Alberta, T6G 2H7, Canada; 2 Department of Medicine, Faculty of Medicine and Dentistry, University of Alberta, Edmonton, Alberta, T6G 2H7, Canada; 3 Signal Transduction Research Group, Faculty of Medicine and Dentistry, University of Alberta, Edmonton, Alberta, T6G 2H7, Canada; Children's Hospital Boston, UNITED STATES

## Abstract

The small GTPase RhoA has been implicated in various cellular activities, including the formation of stress fibers, cell motility, and cytokinesis. In addition to the canonical GTPase cycle, recent findings have suggested that phosphorylation further contributes to the tight regulation of Rho GTPases. Indeed, RhoA is phosphorylated on serine 188 (^188^S) by a number of protein kinases. We have recently reported that Rac1 is phosphorylated on threonine 108 (^108^T) by extracellular signal-regulated kinases (ERK) in response to epidermal growth factor (EGF) stimulation. Here, we provide evidence that RhoA is phosphorylated by ERK on ^88^S and ^100^T in response to EGF stimulation. We show that ERK interacts with RhoA and that this interaction is dependent on the ERK docking site (D-site) at the C-terminus of RhoA. EGF stimulation enhanced the activation of the endogenous RhoA. The phosphomimetic mutant, GFP-RhoA S88E/T100E, when transiently expressed in COS-7 cells, displayed higher GTP-binding than wild type RhoA. Moreover, the expression of GFP-RhoA S88E/T100E increased actin stress fiber formation in COS-7 cells, which is consistent with its higher activity. In contrast to Rac1, phosphorylation of RhoA by ERK does not target RhoA to the nucleus. Finally, we show that regardless of the phosphorylation status of RhoA and Rac1, substitution of the RhoA PBR with the Rac1 PBR targets RhoA to the nucleus and substitution of Rac1 PBR with RhoA PBR significantly reduces the nuclear localization of Rac1. In conclusion, ERK phosphorylates RhoA on ^88^S and ^100^T in response to EGF, which upregulates RhoA activity.

## Introduction

Rho GTPases are monomeric, small GTP-binding proteins belonging to the Ras superfamily. Within the Rho GTPase family, RhoA, Rac1, and Cdc42 have been most extensively characterized [1]. Rho GTPases play pivotal roles in the regulation of cell size, cell proliferation, cell apoptosis, cell polarity, cell adhesion, cell motility and membrane trafficking [2,3]. Like all other small GTP-binding proteins, the regulatory cycle of RhoA is controlled by three distinct families of proteins: guanine nucleotide exchange factors (GEFs) that activate RhoA by promoting uptake of free nucleotide, GTPase-activating proteins (GAPs) that negatively regulate RhoA by stimulating its intrinsic GTPase activity leading to an inactive GDP-bound state, and guanine nucleotide dissociation inhibitors (GDIs) that inhibit the dissociation of GDP from RhoA and prevent the binding of GDP-RhoA to cell membranes. Thus, Rho GEFs, GAPs, and GDIs have been established as the main regulators of Rho GTPases [4]. The GTPase cycle is essential for the biological functions of Rho GTPases, leading to its interaction with downstream effectors [5,6]. It has become evident, however, that a simple GTPase cycle cannot solely explain the variety of functions and signaling initiated by Rho proteins. Recent findings have suggested that additional regulatory mechanisms such as post-transcriptional regulation by microRNAs [7], ubiquitination [8], palmitoylation [9], and phosphorylation [10] might contribute further to the tight regulation of Rho GTPases. Several members of the Rho GTPases have been shown to be regulated by serine, threonine or tyrosine phosphorylation. RhoA was the first Rho GTPase shown to be phosphorylated. RhoA is phosphorylated by cAMP-dependent protein kinase (PKA) and the cGMP-dependent protein kinase (PKG) on serine 188 (^188^S) [6,11–14]. RhoA is also a target for phosphorylation by other kinases such as AMP-activated protein kinase α1 (AMPKα1) and Mst3 kinase [15,16]. RhoA phosphorylation on ^188^S deactivates RhoA by increasing its interaction with RhoGDI, leading to translocation from its site of action at the membrane to the cytosol [5,6,11]. RhoA phosphorylation on ^188^S causes the collapse of actin stress fibers [6,13]. In addition, Cdc42 is phosphorylated on tyrosine 64 (^64^Y) by SRC tyrosine kinase, and this phosphorylation results in the increased interaction between Cdc42 and GDI [17]. RhoE is phosphorylated on serine 11 by ROCK1 and this phosphorylation induces the cytosolic relocation and increased stability of RhoE [18]. Rac1 is phosphorylated on ^71^S by Akt, which does not change Rac1 GTPase activity of Rac1, but inhibits its binding to GTP [19]. Moreover, Rac1 is phosphorylated at ^64^Y by FAK and SRC kinases, potentially playing a role in the regulation of cell spreading [20]. Evidence is accumulating that phosphorylation is playing an increasingly important role in the regulation of Rho GTPase functions.

We have previously shown that extracellular signal-regulated kinases [ERK, consisting of p44 (ERK1) and p42 (ERK2)] phosphorylates ^108^T of Rac1 in response to EGF stimulation [21]. This phosphorylation alters Rac1 activity, its subcellular localization and its role in mediating cell migration. It has been well established that the substrate selectivity of ERKs is dependent on ERK-docking sites (D-sites), with the core consensus motif (K/R)_1-3_-X_1-6_-φ-X-φ (where φ is a hydrophobic residue) located on ERK-interacting proteins [22,23]. We have also shown that the direct interaction between Rac1 and ERK is mediated through the ERK docking site in the Rac1 C-terminus [21]. It is interesting to note that RhoA also contains a putative ERK docking site (D-site) in its C-terminal ^185^KKKSGCLLL^193^.

In the present study, we have investigated whether RhoA and Cdc42 can be phosphorylated by ERK. The results have demonstrated that ^88^S and ^100^T of RhoA are phosphorylated by ERK. There is a direct physical interaction between RhoA and ERK which is dependent on the D-site of RhoA. We also showed that phosphorylation of RhoA by ERK increased its activity and its function in mediating stress fiber formation.

## Materials and Methods

### Cell culture and treatment

COS-7, BT-20, MCF-7, MDA-MB-231, SKBR3 cell lines (purchased from American Type Culture Collection, ATCC) were cultured in DMEM (Dulbecco’s Modified Eagle’s Medium) supplemented with 10% fetal bovine serum (FBS) and antibiotics including penicillin (100U/ml) and streptomycin (100μg/ml). The cells were maintained in a 5% CO2 atmosphere at 37°C. For the EGF treatments, COS-7 cells were incubated with EGF (50 ng/ml) for 15 min or as indicated following serum starvation for 16 h in DMEM medium. For ERK inhibition, cells were pretreated with 5 μM U0126 for 30 min before treating with EGF. For ROCK1 inhibition, COS-7 cells were pretreated with a selective ROCK inhibitor, Y-27632 (5uM), for 60 min before EGF treatment.

### Transient transfection

Plasmid DNA for transfection was prepared by using a Qiagen midiprep kit (Qiagen Inc., Chatsworth, CA) according to the manufacturer’s instructions. COS-7 cells were grown to 70–80% confluence in 6-cm dishes before the transfection. Transfections were performed using the calcium phosphate transfection method using BES buffer [140 mM NaCl, 0.75 mM sodium phosphate dibasic (Na_2_HPO_4_), 25 mM N,N-bis(2-hydroxyethyl)-2-aminoethanesulfonic acid (BES), pH 6.95]. Cells were typically analyzed 48 h post transfection.

### Antibodies and chemicals

Mouse monoclonal (anti-phosphor ERK, p-ERK) and rabbit polyclonal (anti-ERK, anti-RhoA, anti-lamin A, anti-ROCK1, anti-mDia, anti-α-tubulin, anti-E-cadherin) and goat polyclonal anti-p-MYPT1(Thr 853) antibodies were purchased from Santa Cruz Biotechnology, Inc. (Santa Cruz, CA). Mouse monoclonal anti-threonine, anti-serine antibodies were purchased from Cell Signaling Technology, Inc. (Danvers, MA). Rabbit anti-GFP antibody, was from Clonetech (Mountain View, CA). Purified His tagged RhoA protein was from Cytoskeleton Inc. (Denver, CO). Purified active ERK1 was purchased from SignalChem (Richmond, BC, Canada). U0126, Y-27632, Glutathione cross-linked to 4% agarose, goat anti-mouse IgG conjugated with agarose, protein A conjugated with agarose, and Amino Amido Black staining solution were purchased from Sigma-Aldrich (St. Louis, MO). Unless otherwise specified, all chemicals were purchased from Sigma-Aldrich.

### Plasmids

The glutathione-S-transferase-Rhotekin-RhoA-binding domain (GST-RBD) construct was a gift from Dr. Gary Eitzen (University of Alberta). Constructs including GFP-RhoA, GFP-Rac1, GFP-Rac1T108E, and GST-RhoA had been generated previously in the laboratory [21]. All the mutants with point mutations were created with the QuikChange Multiple Site-directed Mutagenesis Kit (Stratagene, La Jolla, CA) with GFP-RhoA or GST-RhoA as templates. These mutants include GFP-tagged mutant RhoA with mutation of either serine 88 or threonine 100 to alanine, or both sites to alanine (GFP-RhoA S88A, GFP-RhoA T100A, and GFP-RhoA S88A/T100A), GFP-tagged mutant RhoA with mutation of either serine 88 or threonine 100 to glutamic acid, or both sites to glutamic acid (GFP-RhoA S88E, GFP-RhoA T100E, and GFP-RhoA S88E/T100E). We also created a GFP-tagged mutant RhoA with the deletion of ERK D-site (GFP-RhoAΔD), which lacks the 10-amino acid residues from positions 183–192. The GST-tagged RhoA mutants were produced similarly except using GST-RhoA as a template. Plasmids were sequenced to confirm the desired mutations. GFP-Rac1_RhoA-PBR_, which was constructed by replacing the Rac1 PBR (^181^PVKKRKRK^188^) with RhoA PBR (^182^RRGKKKSG^189^), and GFP-RhoA_Rac1-PBR_, which was constructed by replacing RhoA PBR (^182^RRGKKKSG^189^) with Rac1 PBR (^181^PVKKRKRK^188^), were generously provided by Dr. Carol Williams (Medical College of Wisconsin, WI, USA). Constructs encoding GFP-tagged mutant RhoA (PBR Rac1) with the mutation of serine 88 and threonine 100 to glutamic acid (GFP-RhoA S88E/T100E_Rac1-PBR_) and GFP-Rac1 T108E_RhoA-PBR_ were generated by further mutation of ^88^S and ^100^T to E using the method described above, with GFP-Rac1_RhoA-PBR_ and GST-RhoA_Rac1-PBR_ as templates.

### Expression and purification of GST-fusion proteins

The purification of various GST-fusion proteins were performed as previously described [21]. Briefly, pGEX plasmids encoding GST alone, wild type GST-RhoA or mutant GST-RhoA, and GST-RBD were transformed into *Escherichia coli* DH5α. The GST-fusion proteins were purified with glutathione-sepharose beads. The purified fusion proteins that were immobilized on beads were used for GST pull-down assays, and eluted GST, wild type GST-RhoA, and mutant proteins were used for *in vitro* kinase assays.

### GST pull-down assay

COS-7 cells were treated with or without EGF and then lysed into BOS buffer (50 mM Tris-HCl, pH 7.4, 200 mM NaCl, 1% Nonidet P-40, 10% glycerol, 10 mM NaF, 2.5 mM MgCl_2_, and 1 mM EDTA) with protease inhibitors. The lysates were centrifuged at 21,000 × *g* at 4°C for 15 min. Supernatants were used in the pull-down assays. GST-fusion proteins bound to glutathione-sepharose beads were added to the supernatant and incubated at 4°C for 2 h with gentle shaking. Beads were collected by centrifugation and washed three times with BOS buffer after which 2x sample loading buffer was added to elute the bound proteins. The pulled-down proteins were resolved by SDS-PAGE and analyzed by immunoblotting.

### RhoA activation assay

RhoA activation was determined by using an assay developed by Ren and Schwartz [24]. The RhoA binding domain of Rhotekin, a RhoA effector, was used as a GST fusion protein to pull down active RhoA. Briefly, COS-7 cells, either transfected or not transfected with expression constructs encoding GFP-RhoA (wild type or mutants), were lysed in BOS buffer (50 mM Tris-HCl, pH 7.6, 150 mM NaCl, 1% Triton X-100, and 10 mM MgCl_2_) with protease inhibitors. The lysates were centrifuged at 21,000 × *g* at 4°C for 15 min. Supernatants were used in the binding assay. GST-RBD fusion proteins bound to glutathione-sepharose-beads in PBS were added to the supernatants followed by incubation at 4°C for 2 h with gentle agitation. Beads were collected by centrifugation, washed three times with BOS buffer, after which SDS sample loading buffer was added. The pulled-down active, GTP-bound RhoA was resolved by SDS-PAGE and the activity was analyzed by immunoblotting with anti-GFP or anti-Rho antibodies.

### *In vitro* ERK kinase assay

GST-RhoA, GST-RhoA S88A, GST-RhoA T100A, GST-RhoA S88A/T100A, GST-RhoA S88E, GST-RhoA T100E, and GST-RhoA S88E/T100E purified proteins were eluted from the glutathione-sepharose beads using glutathione elution buffer (10 mM reduced glutathione and 50 mM Tris-HCl, pH 8.0). Approximately 2 μg of GST fusion proteins or 5 μg of purified His-tagged RhoA, Rac1, and Cdc42 proteins were incubated with 0.1 μg of active ERK1 protein in kinase buffer (5 mM MOPS, pH 7.2, 2.5 mM β-glycerophosphate, 5 mM MgCl_2_, 1 mM EGTA, 0.4 mM EDTA, 0.05 mM dithiothreitol) in the presence of 200 μM ATP and 5 μCi of [γ-^32^P]ATP at 30°C for 60 min in a volume of 25 μl. Reactions were stopped by adding SDS-PAGE sample loading buffer and boiling for 5 min. Proteins in these samples were then separated by SDS-PAGE (8% gel), transferred to a polyvinylidene difluoride membrane (PVDF), and subjected to autoradiography.

### Immunoprecipitation

Immunoprecipitation (IP) experiments were carried out as described previously [25]. Briefly, cells were lysed with IP buffer [20 mM Tris, pH 7.5, 150 mM NaCl, 1% Nonidet P-40, 0.1% sodium deoxycholate, 100 mm NaF, 5 mM MgCl_2_, 0.5 mM Na_3_VO_4_, 0.02% NaN_3_, 0.1 mM 4-(2-aminoethyl)-benzenesulfonyl fluoride, 10 μg/ml aprotinin, and 1 μM pepstatin A]. Cell lysates were centrifuged at 21,000 × *g* for 15 min to remove debris. The supernatants, containing approximately 1 mg of total protein, were pre-cleared with agarose beads and then were incubated with 1 μg of specific antibody at 4°C overnight. Secondary antibodies or protein A conjugated with agarose was then added to each supernatant/antibody mixture. Following 2 h incubation at 4°C with agitation, the supernatant/antibody mixture were centrifuged and the pelleted agarose beads and the non-precipitated supernatant were collected. The agarose beads were washed three times with IP buffer, and then mixed with 2x sample loading buffer. The sample was boiled for 5 min and subjected to SDS-PAGE followed by immunoblotting.

### Immunoblotting

The protein concentration of cell lysates was examined by Bradford analysis. Protein samples (20 μg protein for each sample) were resolved by SDS-PAGE and transferred onto nitrocellulose or PVDF membranes. After blocking in 3% non-fat dry milk in Tris-buffered saline for 60 min, membranes were incubated with primary antibody at 4°C overnight with gentle agitation. The membranes were washed, and the primary antibodies were detected by incubation in their corresponding horseradish peroxidase-conjugated secondary antibodies. After washing the membranes, the blots were analyzed by enhanced chemiluminescence development and light detection with Fuji Super RX film.

### Subcellular fractionation

For COS-7 cells expressing GFP-tagged RhoA (wild type and mutants), the cell homogenates were separated into two fractions: the nuclear faction and the non-nuclear fraction, and fractionation was performed as we have described previously [26]. Briefly, COS-7 cells were treated with or without EGF (50 ng/ml) and scraped into homogenization buffer [0.25 M sucrose, 20 mM Tris-HCl, pH 7.0, 1 mM MgCl_2_, 4 mM NaF, 0.5 mM Na_3_VO_4_, 0.1 mM 4-(2-aminoethyl)-benzenesulfonyl fluoride, 10 μg/ml aprotinin, and 1 μM pepstatin A]. Following the homogenization with a dounce homogenizer, the lysate was passed through a 25G needle 10 times. The nuclei were then pelleted from the homogenate by centrifugation at 200 × *g* for 10 min twice. The supernatant was then centrifuged at 14,000 × *g* for 10 min to pellet the contaminating nuclei and cell debris. The supernatant contained the cytoplasm and cell membrane. The pellet from the first centrifugation was suspended in homogenization buffer and then centrifuged at 200 × *g* for 10 min at least 3 times to remove cytoplasmic contamination. The pellets were then suspended in M-Per and used as nuclear fractions. The loading volumes of the nuclear fraction and non-nuclear fraction were about 25%, and 3% of total sample volume, respectively, and were analyzed by SDS-PAGE followed by immunoblotting.

To locate the endogenous RhoA, the homogenates of COS-7 cells were subjected to subcellular fractionation to yield nuclear, total membrane, and cytosolic fractions. Briefly, COS-7 cells were treated with EGF (50 ng/ml) for the durations indicated in the figure, and the nuclear fraction was obtained as described above. The postnuclear supernatant was centrifuged at 100,000 × *g* for 30 min to yield a supernatant which was collected as the cytosolic fraction, and the pellet was resuspended in 2x SDS-PAGE loading buffer and collected as the total membrane fraction.

### Subcellular localization of proteins by fluorescence microscopy

Subcellular localization of GFP-tagged proteins were examined by fluorescence microscopy as previously described. COS-7 cells grown on glass cover slips were transfected with various GFP-tagged proteins for 48 h. Following serum starvation for 12 h, the cells were treated with 50 ng/ml EGF for 15 min. The cells were then fixed with 4% paraformaldehyde. The cells were examined for GFP-tagged proteins with an inverted fluorescence microscope (Axiovert 200; Carl Zeiss, Inc. Germany) with a Plan-Apochromat 63×/1.40 oil immersion objective equipped with a digital CCD camera and using Northern Eclipse software (Empix Imaging, Inc. Canada). The subcellular localization of GFP-tagged proteins was identified visually. To quantify nuclear localization of the GFP-tagged proteins, we counted at least 20 transfected cells for each experiment and each data point is the average of three experiments with more than 60 transfected cells.

### Immunofluorescence staining for stress fibers

COS-7 cells were grown on glass cover slips and transiently transfected with expression constructs encoding wild type or mutant GFP-RhoA proteins. After serum starvation for 16 h, cells were treated with 50 ng/ml EGF for the indicated duration. The cells were fixed by immersion in 4% paraformaldehyde in PBS for 5 min. After removal of paraformaldehyde and washing with PBS, the cells were permeabilized with 0.5% Triton X-100 in PBS for 10 min at room temperature. Then, the cells on the cover slips were incubated with 70 nM rhodamine-conjugated phalloidin for 30 min at room temperature. The stained cells were analyzed by Delta Vision Deconvolution microscopic systems (Applied Precision, Issaquah, WA). Photographs were taken with a digital camera by superimposing the monochrome graphs of two channels, and the data were analyzed using DeltaVision SoftWoRx software. To quantify the stress fiber formation, the boundary of the cells was determined by using differential interference contrast images, after which the total intensity of the phalloidin fluorescence was calculated by Delta Vision SoftWoRx software and used as a measure of stress fiber formation. Each value is the mean of at least three experiments.

### Statistical analysis

Statistical analysis was performed using one-way analysis of variance (ANOVA) followed by Tukey’s Post Hoc test in SPSS 17.0 (SPSS Inc., Chicago, IL). Significant differences were considered when *p* <0.05 or less.

## Results

### The direct interaction between RhoA and ERK is mediated by the ERK docking site in the C-terminus of RhoA

**Fig 1:**
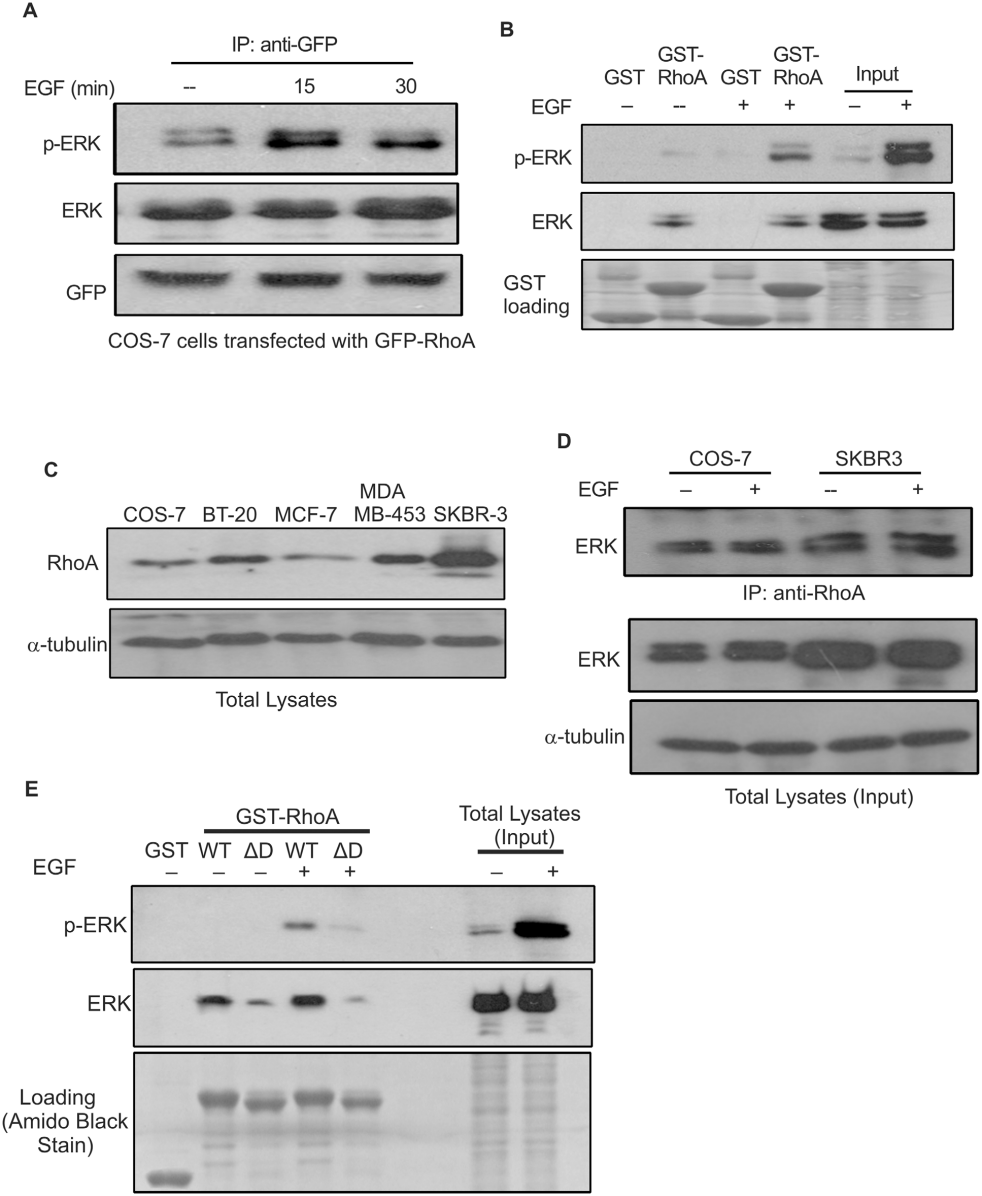
Interaction between RhoA and ERK is mediated by the RhoA D-site. **(A)** Co-immunoprecipitation of ERK and p-ERK with RhoA. COS-7 cells expressing GFP-RhoA were stimulated with EGF as indicated. GFP-RhoA was IPed from cell lysates with antibodies to GFP, and the co-IPed ERK and phosphor ERK (p-ERK) were analyzed by immunoblotting with antibodies to ERK and p-ERK. **(B)** Interaction between ERK and GST-RhoA. Lysates of COS-7 cells, with or without EGF stimulation, were incubated with GST-RhoA or GST bound to glutathione sepharose beads. The sepharose beads were collected, washed and analyzed by immunoblotting with antibodies against p-ERK and ERK. GST/GST-RhoA fusion protein loading was verified by Amido Black staining of the nitrocellulose membrane. **(C)** The expression levels of endogenous RhoA in COS-7 cells and various breast cancer cell lines. Cells were lysed and the expression levels of RhoA were determined by immunoblotting with anti-RhoA antibody. α-tubulin was used as a loading control. **(D)** Interaction between endogenous RhoA and ERK in COS-7 and SKBR3 cells. Endogenous RhoA was IPed from lysates of COS-7 and SKBR3 cells by anti-RhoA antibody, and the co-IP of endogenous ERK was determined by immunoblotting with antibodies to ERK. α-tubulin was used as a loading control. **(E)** The effect of the RhoA D-site on the interaction between RhoA and ERK. Lysates of COS-7 cells (with or without EGF stimulation) were incubated with GST-RhoA or mutant GST-RhoA with its D-site deleted (GST-RhoAΔD) bound to glutathione agarose beads. The sepharose beads were then collected, washed and analyzed by immunoblotting with antibodies against p-ERK and ERK. GST/GST fusion protein loading was verified by Amido Black stain of the nitrocellulose membrane.

We have shown previously that Rac1 is directly associated with ERK, but in an EGF-independent manner [21]. In this study, we first examined whether RhoA also interacts with ERK. We expressed GFP-RhoA in COS-7 cells by transient transfection. Then, we immunoprecipitated (IPed) GFP-RhoA and immunoblotted the immunoprecipitates with antibodies to ERK and phosphorylated ERK (p-ERK). As shown in Fig 1, ERK was co-IPed with GFP-RhoA with or without EGF stimulation. However, the association of RhoA with p-ERK was much stronger following EGF stimulation, which could be due to the increase of p-ERK in response to EGF (Fig 1A). We further examined the interaction between RhoA and ERK by GST pull-down assay (Fig 1B). Total cell lysates from COS-7 cells, either treated with EGF or not treated, were incubated with GST-RhoA. ERK was associated with GST-RhoA with or without EGF stimulation; however, the association between p-ERK and GST-RhoA was only observed following EGF stimulation (Fig 1B). As a control, we showed that there is no association between ERK or p-ERK and GST (Fig 1B).

We next examined whether endogenous RhoA and ERK interact with each other and whether this association also exists in breast cancer cell lines. We examined the expression level of RhoA in COS-7 cells and various breast cancer cells including BT-20, MCF-7, MDA-MB-453, and SKBR-3 cells. As shown in Fig 1C, RhoA was well expressed in all of these cells. Except for MCF-7 cells, the expression level of RhoA was notably higher in the rest of the breast cancer cells than in COS-7 cells, with highest expression level in SKBR-3 cells (Fig 1C). The interaction between endogenous RhoA and ERK was examined by co-IP experiments in COS-7 and SKBR-3 cells (Fig 1D). Following the IP with antibodies to RhoA, the immunoprecipitates were immunoblotted with antibodies to ERK. We showed that ERK co-IPed with endogenous RhoA with or without EGF stimulation in both COS-7 and SKBR-3 cells (Fig 1D).

It has been well established that the substrate selectivity of ERK is dependent on ERK-docking sites (D-sites) [22,23]. The amino acid sequence analysis indicates that RhoA contains a putative ERK D-site ^185^KKKSGCLLL^193^ in its C-terminus. We examined whether the interaction between RhoA and ERK is mediated by this putative RhoA D-site. We constructed a GST fusion RhoA mutant with the deletion of its putative D-site (GST-RhoAΔD) and examined its interaction with ERK by GST pull-down assay (Fig 1E). Our results showed that GST-RhoAΔD pulled down substantially less p-ERK and total ERK compared with wild type GST-RhoA, indicating that the D-site is required for the interaction between RhoA and ERK.

### Phosphorylation of RhoA by ERK in response to EGF treatment

**Fig 2:**
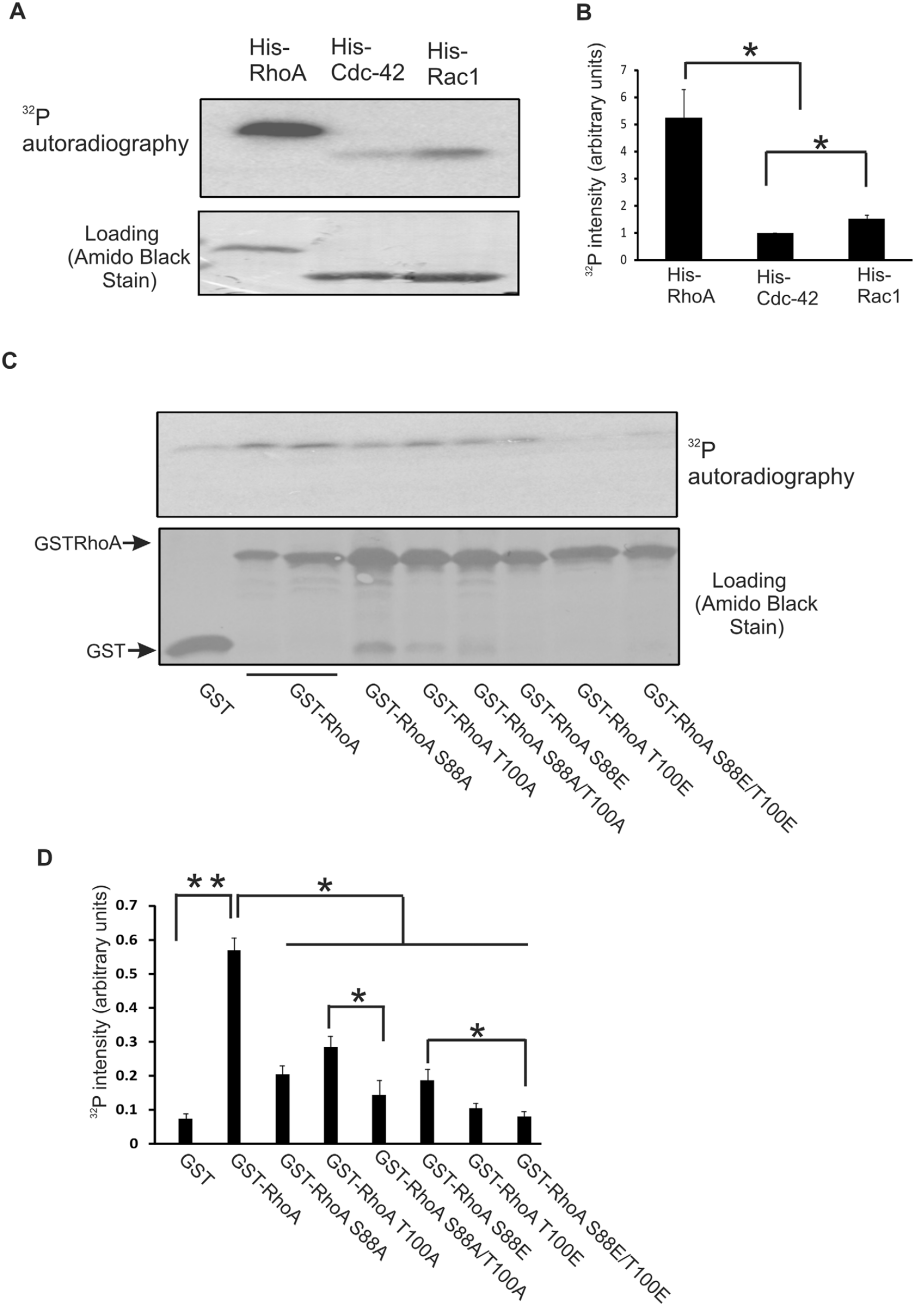
Phosphorylation of RhoA on ^88^S and ^100^T by active ERK1 *in vitro*. **(A)** Phosphorylation of His-RhoA, His-Cdc42, and His-Rac1 by ERK1 *in vitro*. The phosphorylation of purified His-tagged Rho proteins by purified active ERK1 was performed with an *in vitro* ERK kinase assay kit in the presence of [γ-^32^P]ATP as described in the Materials and Methods. ^32^P was detected by autoradiography. **(B)** Quantification of the data from three independent experiments as described in (A). The intensity of the bands of ^32^P was normalized against the intensity of the His-tagged protein loading. The error bar is standard error. * indicates p<0.05. **(C)** Phosphorylation of GST-RhoA and mutant proteins by purified ERK1 *in vitro*. The phosphorylation of GST-RhoA (5 μg) and various mutant GST-RhoA (5 μg) by purified active ERK1 was performed as described in (A). GST was used as a negative control. GST fusion protein loading was verified by Amido Black stain of the PVDF membrane. **(D)** Quantification of the data from three independent experiments as described in panel C. The intensity of the bands of ^32^P was normalized against the intensity of the GST fusion protein loading. The error bar is standard error. * indicates p<0.05 and ** indicates p<0.01.

We have shown previously that the interaction between Rac1 and ERK resulted in Rac1 phosphorylation on T108 [21]. In the present study, we examined whether ERK also phosphorylates RhoA and Cdc42. Using affinity-purified recombinant His-tagged fusion proteins, we showed by *in vitro* ERK kinase assay that RhoA, Rac1, and Cdc42 were all phosphorylated by the activated ERK1. Interestingly, RhoA phosphorylation was much stronger than Rac1, and Cdc42 phosphorylation was very weak (Fig 2A & 2B).

As a proline directed serine/threonine protein kinase, ERK phosphorylates the serine or threonine in the dipeptide motif S/T-P [27]. Analysis of the RhoA protein sequence showed ^88^SP and ^100^TP could be two potential ERK phosphorylation sites. To determine which site is phosphorylated by ERK, we generated several RhoA mutants with the substitution of ^88^S and/or ^100^T with alanine (A) or glutamic acid (E). These mutants include: GST-RhoA S88A, GST-RhoA T100A, GST-RhoA S88A/T100A, GST-RhoA S88E, GST-RhoA T100E, and GST-RhoA S88E/T100E. The S or T to E mutants are phosphomimetic mutants. We then examined whether these mutants were phosphorylated by using the *in vitro* ERK kinase assay as described in Materials and Methods. As shown in Fig 2C & 2D, the results were complicated. In general, mutation of either ^88^S or ^100^T to E significantly reduced RhoA phosphorylation by ERK. Simultaneous mutation of both ^88^S and ^100^T inhibited RhoA phosphorylation more strongly than the single mutation of either ^88^S or ^100^T, except for the mutation of ^100^T to ^100^E that inhibited RhoA phosphorylation to the same degree as the double mutation of both ^88^S and ^100^T to E. These results suggest that both ^88^SP and ^100^TP are phosphorylated by ERK in response to EGF.

**Fig 3:**
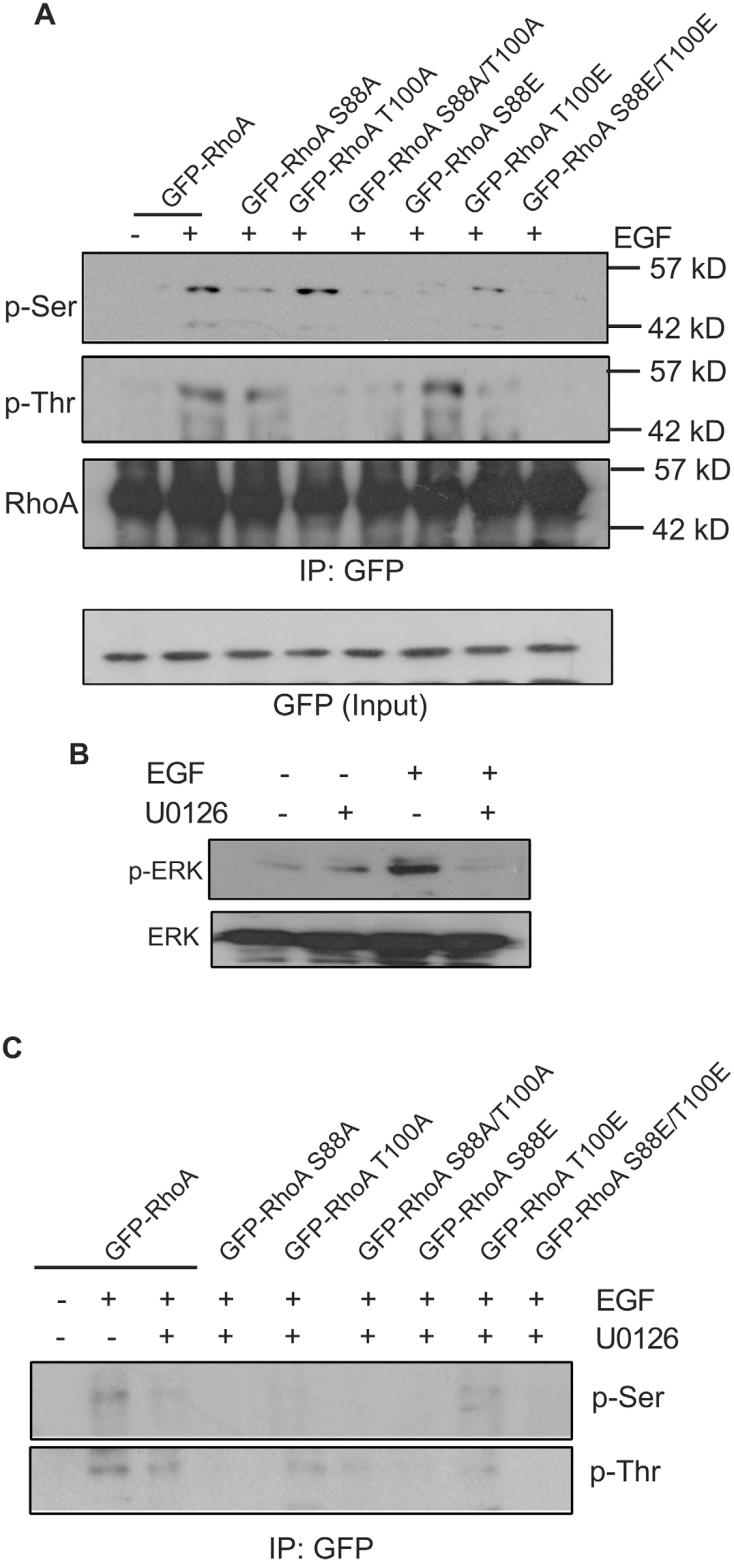
Phosphorylation of RhoA on ^88^S and ^100^T by ERK in response to EGF *in vivo*. **(A)** EGF-induced serine and threonine phosphorylation of RhoA and mutants. COS-7 cells were transfected with expression constructs encoding GFP-RhoA and various GFP-RhoA mutants. The cells were stimulated with EGF (50 ng/ml). GFP-RhoA/RhoA mutant proteins were immunoprecipitated with anti-GFP antibodies and the serine or threonine phosphorylation of GFP-RhoA was detected by immunoblotting with anti-p-Ser or anti-p-Thr antibodies. To confirm the GFP antibody captured protein is GFP-RhoA, the membranes with p-Ser (shown in the panel) and p-Thr blots were reprobed with anti-RhoA antibody. The expression level of GFP-tagged RhoA proteins was determined by immunoblotting of the total lysates with antibody to GFP (bottom panel). **(B)** The effects of MEK inhibitor U0126 on EGF-induced phosphorylation of ERK. **(C)** The effects of MEK inhibitor U0126 on EGF-induced phosphorylation of RhoA proteins. The COS-7 cells were treated similar to the method described in (A), but with the addition of U0126. Following IP of cell lysates with anti-GFP antibodies, the phosphorylation of GFP-RhoA and its various mutants was determined by immunoblotting with antibodies to p-Ser and p-Thr.

We further examined whether ^88^SP and ^100^TP are ERK phosphorylation sites by immunoblotting. We constructed a series of GFP-tagged RhoA mutants including GFP-RhoA S88A, GFP-RhoA T100A, GFP-RhoA S88A/T100A, GFP-RhoA S88E, GFP-RhoA T100E, and GFP-RhoA S88E/T100E. These mutants were expressed in COS-7 cells by transient transfection. Following EGF stimulation for 15 min, these RhoA mutants were IPed by anti-GFP antibody and the phosphorylation status of these mutants was examined with anti-phosphoserine (p-Ser) or anti-phosphothreonine (p-Thr) antibodies. As shown in Fig 3A, EGF treatment induced RhoA phosphorylation that can be detected by both p-Ser and p-Thr antibodies. Mutation of ^88^S substantially reduced the phosphorylation level of RhoA serine phosphorylation, but had no effect on the threonine phosphorylation of RhoA. Similarly, mutation of ^100^T strongly inhibited RhoA threonine phosphorylation, but had no effect on RhoA serine phosphorylation. Mutation of both ^88^S and ^100^T simultaneously inhibits EGF-induced serine and threonine phosphorylation of RhoA. These results confirmed that both RhoA ^88^S and ^100^T are phosphorylated in response to EGF.

We next examined whether EGF-induced RhoA phosphorylation is mediated by ERK *in vivo*. As a positive control, we verified that U0126, a MEK inhibitor, blocked EGF-induced ERK phosphorylation in COS-7 cells (Fig 3B). We then expressed GFP-RhoA and various mutants in COS-7 and stimulated the cells with EGF in the presence of U0126. Following IP of these expressed GFP-tagged proteins, the phosphorylation of these proteins was examined by immunoblotting with antibodies to p-Ser and p-Thr. As shown in Fig 3C, inhibition of ERK by U0126 blocked EGF-induced phosphorylation of wild type and mutant RhoA, which suggested that EGF-induced RhoA phosphorylation of both ^88^S and ^100^T is most likely mediated by ERK.

### The effects of RhoA phosphorylation on its interaction with ERK

**Fig 4:**
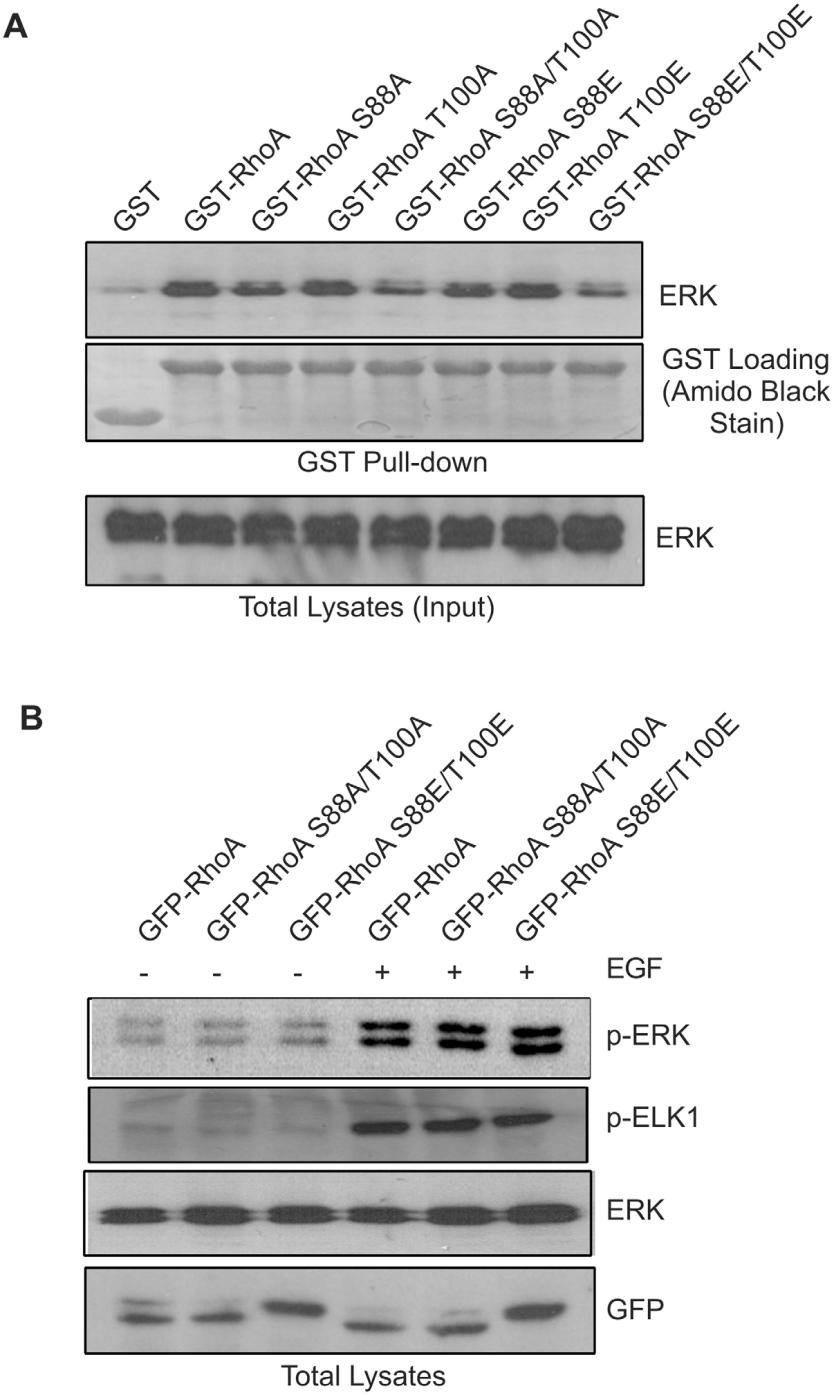
The effects of RhoA phosphorylation (^88^S and ^100^T) on RhoA interaction with ERK and on EGF-induced ERK phosphorylation. **(A)** The effects of RhoA phosphorylation on its interaction with ERK. COS-7 cells were serum starved and treated with EGF (50 ng/ml) for 15 min. The cell lysates were incubated with GST-fused wild type and mutant RhoA proteins bound to glutathione-sepharose beads. ERK pulldowns were analyzed by immunoblotting with antibodies to ERK. GST fusion protein loading was verified by Amido Black staining of the nitrocellulose membrane. **(B)** The effects of RhoA phosphorylation on EGF-induced ERK phosphorylation. COS-7 cells were transfected with expression constructs encoding GFP-tagged wild type and mutant RhoA proteins and these proteins were overexpressed. After serum starvation, cells were stimulated with EGF for 15 min. ERK phosphorylation and activation was determined by immunoblotting cell lysates with antibodies to p-ERK and p-ELK1, respectively. The expression of GFP-RhoA wild type and mutant proteins was determined by immunoblotting with antibodies to GFP.

We showed above that the interaction between RhoA and ERK is mediated by RhoA D-site (Fig 1E). We next examined whether the phosphorylation status of RhoA affects its interaction with ERK. By using GST pull down assays, we showed that all of the RhoA single mutants including GST-RhoA S88A, S88E, T100A, and T100E bind to ERK similarly as wild type RhoA (Fig 4A). However, the double RhoA mutants, including both the alanine mutant that are unable to be phosphorylated by ERK (GST-RhoA S88A/T100A) and phosphomimetic RhoA mutant (GST-RhoA S88E/T100E), bind to ERK at a much lower level than the wild type RhoA (Fig 4A). These data indicated that RhoA and ERK interaction is mediated mostly by RhoA D-site, but is also affected by the ERK phosphorylation sites S88 and T100.

It has been reported that RhoA activation regulates ERK activity, although the data are controversial with regard to the net effects of this regulation [28,29]. We examined the effects of RhoA phosphorylation on the activation of ERK. We examined whether overexpression of GFP-RhoA-S88A/T100A or GFP-RhoA-S88E/T100E in COS-7 cells affects ERK activation. We showed that overexpression of GFP-RhoA S88A/T100A or GFP-RhoA S88E/T100E did not have any detectable effects on EGF-induced ERK phosphorylation (Fig 4B). Moreover, the EGF-induced activation of Elk1, an ERK substrate, was also not affected by RhoA phosphorylation on S88 and T100 (Fig 4B).

### The effects of RhoA phosphorylation on RhoA activity

We next examined whether phosphorylation by ERK regulates RhoA activity. Since only the activated RhoA is able to bind the Rho-binding domain (RBD; amino acids 7–89) of Rhotekin, the activation of RhoA was assessed by its binding to the GST fusion RBD of Rhotekin (GST-RBD).

**Fig 5:**
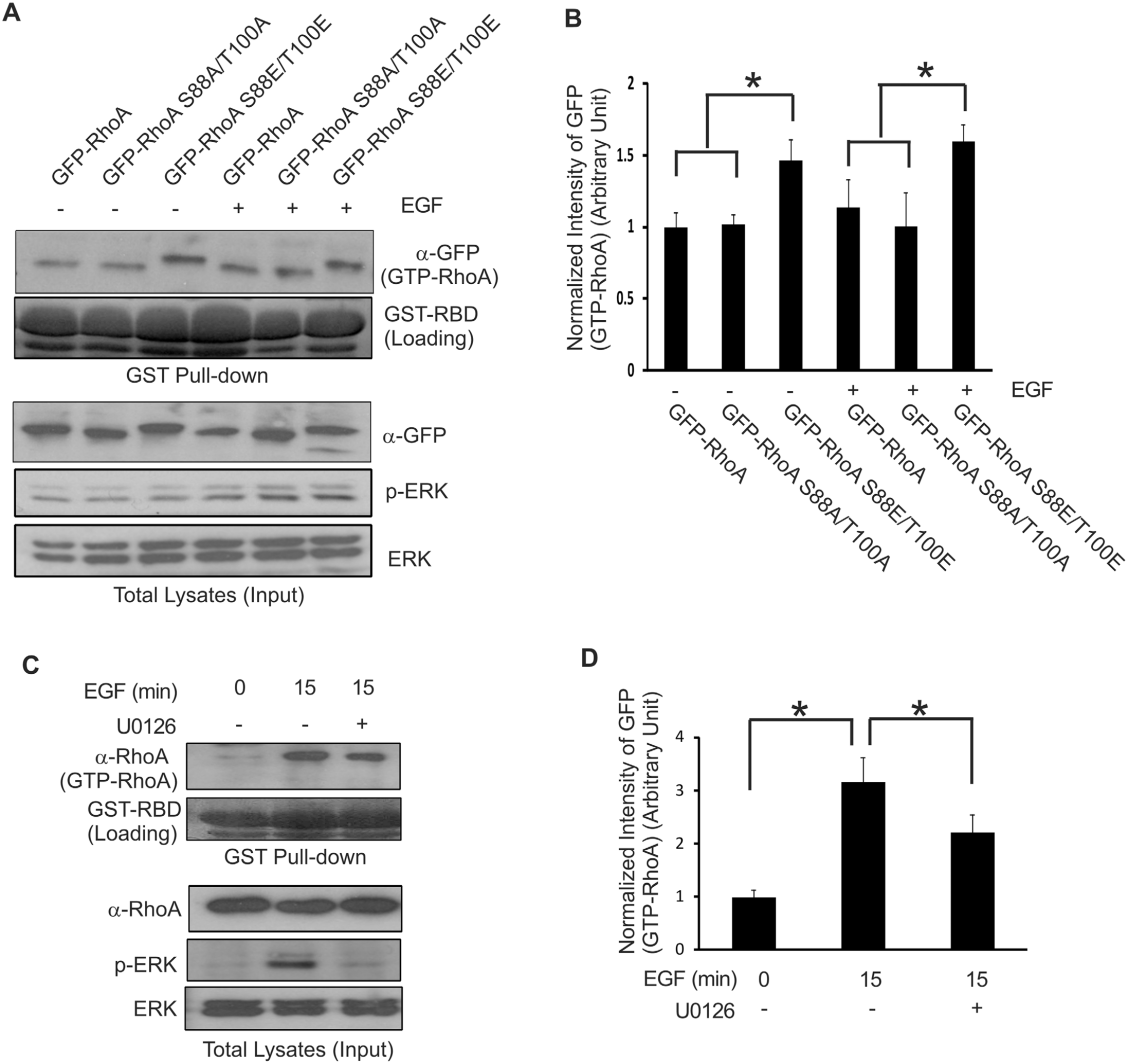
The effects of RhoA phosphorylation on the activation of RhoA. **(A)** The activity of wild type and various mutant RhoA proteins in response to EGF. COS-7 cells were transfected with expression constructs encoding wild type and mutant GFP-tagged RhoA proteins. After serum starvation, cells were stimulated with EGF for 15 min. Cell lysates were incubated with a GST fusion Rhotekin Rho-binding domain (GST-RBD). The active RhoA proteins that bound to GST-RBD were determined by immunoblotting with antibodies to GFP. **(B)** Quantification of the data from (A). The GTP-GFP-RhoA protein intensity was normalized to the intensity of the expressed GFP proteins (input) as detected by anti-GFP antibodies. **(C)** The effects of U0126 on the activation of endogenous RhoA in response to EGF. COS-7 cells were stimulated with EGF for the indicated time with or without U0126. The amount of active RhoA was determined by GST-RBD pull down assay as described in (A), except that antibodies to RhoA were used to detect the endogenous RhoA. **(D)** Quantification of the data from (C). The GTP-RhoA protein intensity was normalized to the intensity of the total endogenous RhoA protein (input) as detected by anti-RhoA antibodies. Each value is the average of at least three experiments and the error bar is standard error. * indicates p<0.05.

We expressed GFP-RhoA S88E/T100E and GFP-RhoA S88A/T100A in COS-7 cells by transient transfection. The cells were either not treated or stimulated with EGF for 15 min and the cell lysates were incubated with GST-RBD. The pulled-down GFP-tagged RhoA and mutants were examined by immunoblotting with antibody to GFP. As shown in Fig 5A & 5B, GST-RBD was able to pull down a larger amount of phosphomimetic GFP-RhoA S88E/T100E than wild type GFP-RhoA and the mutant GFP-RhoA S88A/T100A with or without EGF stimulation. Although the increase in active, wild type GFP-RhoA after EGF treatment is marginal, the significantly higher levels of active GFP-RhoA S88E/T100E (regardless of EGF stimulation) suggest that the phosphorylation of these two sites is important in RhoA activation.

We also examined whether ERK activation is required for EGF-induced activation of endogenous RhoA. We stimulated the cells with EGF with or without U0126. The activated GTP-bound RhoA was pulled down with GST-RBD of Rhotekin. We showed that EGF stimulated RhoA activation and this activation was inhibited by U0126 (Fig 5C & 5D). As controls, we showed that EGF stimulated ERK activation and this ERK activation is abolished in the presence of U0126 (Fig 5C). Together our results indicate that phosphorylation of RhoA ^88^S and ^100^T by ERK in response to EGF enhances RhoA activity.

### The effects of RhoA phosphorylation on cell stress fiber formation

**Fig 6:**
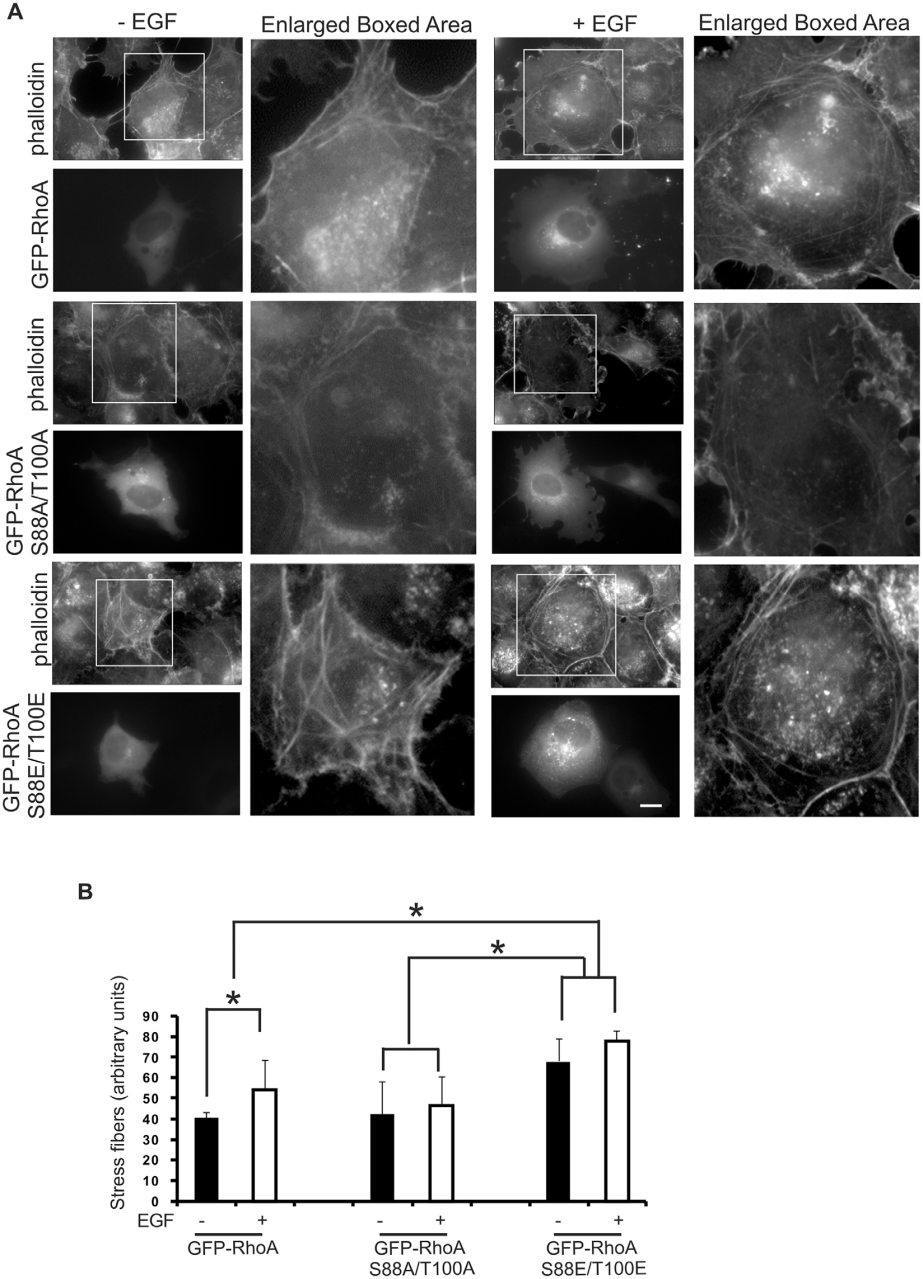
The effects of EGF and RhoA phosphorylation on actin stress fiber formation in COS-7 cells. **(A)** Images of actin stress fibers. COS-7 cells were transfected with expression constructs encoding GFP-tagged wild type, 88A/100A (S88A/T100A) or 88E/100E (S88E/T100E) RhoA. The formation of actin stress fibers was viewed by fluorescence microscopy following staining with 70 nM rhodamine-conjugated phalloidin as described in the Materials and Methods. Boxed areas are shown at higher magnification. Size bar = 20 μm. **(B)** Quantification of the stress fibers was as described in the Materials and Methods. Each value is the mean of at least three experiments with more than 20 cells analyzed for each experiment. The error bar is standard error. * indicates p<0.05.

The most well-studied function of RhoA is its capacity to regulate the formation of actin stress fibers, which consist of long bundles of filaments traversing the cell [30]. EGF has also been shown to stimulate the formation of actin stress fibers [30–32], and this stimulation is mediated by RhoA [30]. To determine the role of RhoA ^88^S and ^100^T phosphorylation in EGF-induced formation of actin stress fibers, we overexpressed GFP-RhoA, GFP-RhoA S88A/T100A and GFP-RhoA S88E/T100E in COS-7 cells by transient transfection. The organization of stress fibers was observed using phalloidin staining. Our results showed that cells with the overexpression of either GFP-RhoA or GFP-RhoA S88E/T100E had enhanced stress fiber formation when compared with non-transfected cells (Fig 6A). Cells with overexpression of GFP-RhoA S88E/T100E showed the strongest phalloidin staining with or without EGF stimulation (Fig 6A & 6B). Treatment with EGF for 15 min increased the intensity of the stress fibers in cells transfected with wild type GFP-RhoA (Fig 6A & 6B). However, overexpression of GFP-RhoA S88A/T100A had little effect on the formation of stress fibers (Fig 6A & 6B). These results indicated that RhoA phosphorylation by ERK enhanced its function in regulating the formation of stress fibers, possible through increasing RhoA activity.

**Fig 7:**
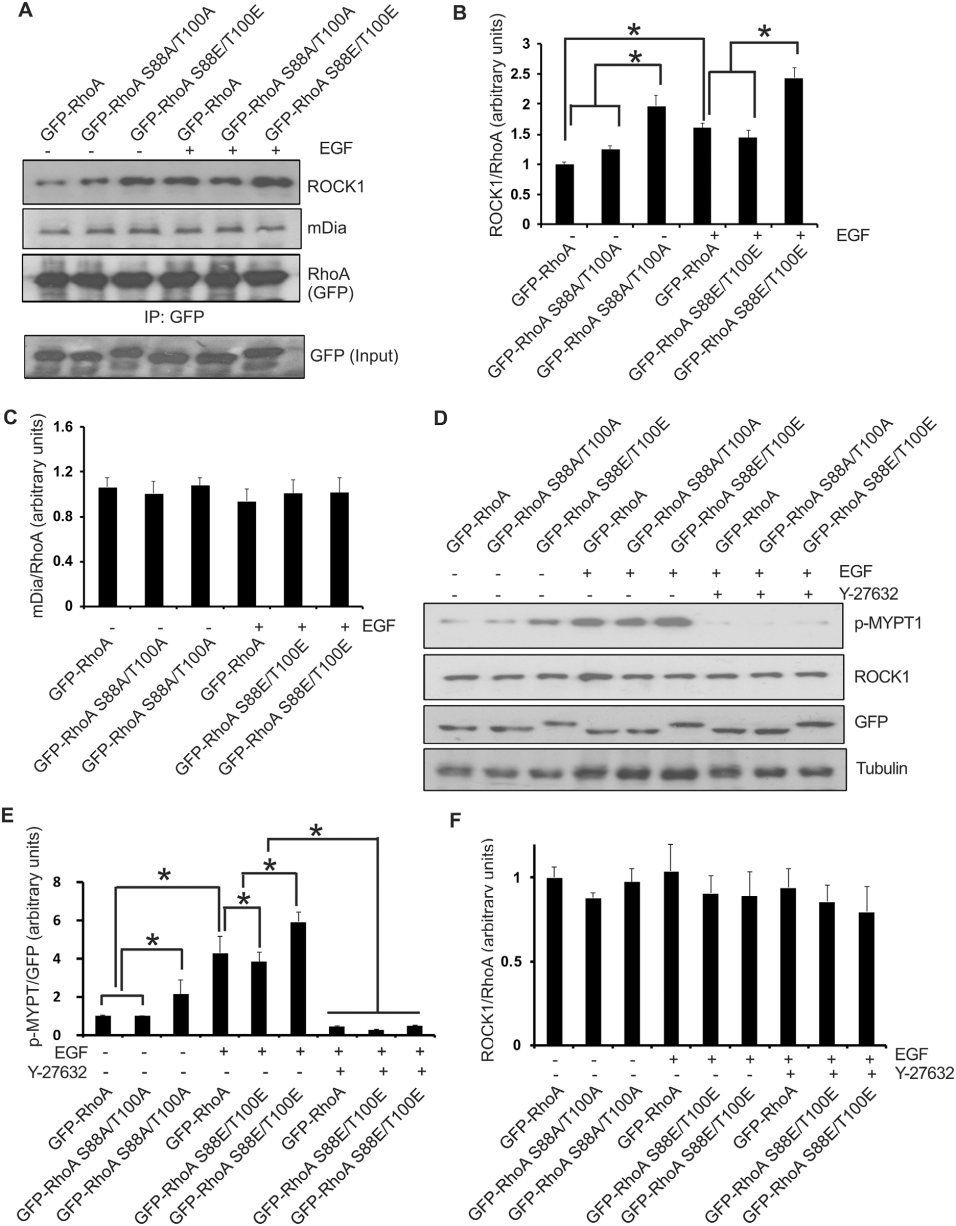
The effects of RhoA phosphorylation on its interaction with ROCK1 and mDia. **(A)** Co-IP of wild type and mutant RhoA with ROCK1/mDia. COS-7 cells were transfected with constructs encoding wild type or mutant GFP-tagged RhoA and stimulated with EGF (50 ng/ml). GFP-tagged wild type and mutant RhoA proteins were immunoprecipitated from cell lysates with anti-GFP antibodies and the co-IPed ROCK1 and mDia were detected with anti-ROCK1 and anti-mDia antibodies. The input GFP was determined by immunoblotting the whole lysate with anti-GFP antibodies (bottom panel). (**B&C**) Quantification of the co-IPed ROCK1 (B) and mDia (C). The binding between ROCK1/mDia and the RhoA proteins was measured as the ratio of the ROCK1/mDia band intensity relative to the RhoA band intensity. Each value is the mean of at least three experiments. The error bar is standard error. * indicates p<0.05. (**D**) The effects of RhoA phosphorylation on ROCK1 activity. COS-7 cells were transfected with wild type or mutant GFP tagged RhoA. After pretreatment with Y-27632 (5uM) for 60 min, cells were stimulated with EGF (50 ng/ml) for 15min. (**E&F**) Quantification of the phosphorylation level of MYPT1 (E) and ROCK1protein (F) measured by the ratio between p-MYPT1 and ROCK1band intensity relative to GFP band intensity. Each value is the mean of at least three experiments. The error bar is standard error. * indicates p<0.05.

To understand the mechanism by which RhoA phosphorylation increases stress fiber formation, we examined whether RhoA phosphorylation increases its interaction with its effectors, ROCK1 and mDia. Several RhoA substrates including ROCK1 and mDia have been implicated in mediating RhoA regulation of actin remodeling [33–35]. We expressed GFP-RhoA, GFP-RhoA S88A/T100A and GFP-RhoA S88E/T100E in COS-7 cells by transient transfection. The cells were either not treated or treated with EGF for 15 min. GFP-tagged RhoA and the mutants were IPed with antibody to GFP. The co-IP of ROCK1 and mDia was determined by immunoblotting. As shown in Fig 7A–7C, EGF stimulates the interaction between ROCK1 and wild type RhoA. The phosphomimetic GFP-RhoA S88E/T100E strongly interacts with ROCK1 with or without EGF stimulation. The interaction between GFP-RhoA S88A/T100A and ROCK1 is weaker (Fig 7A & 7B). These results suggest that the phosphorylation of RhoA ^88^S and ^100^T increases the interaction between RhoA and ROCK1. It has been reported that ROCK1 is activated when it binds to RhoA, and ROCK1 promotes the formation of actin stress fibers and adhesion complexes [36–38]. In contrast, the interaction between mDia and RhoA is not affected by EGF stimulation and the phosphorylation status of RhoA (Fig 7A & 7C).

To determine if ROCK1 activity has changed, we investigated the phosphorylation level of MYPT1, a substrate of ROCK1 [39]. As shown in Fig 7D–7F, EGF treatment did not affect ROCK1 protein expression level; however, it increased MYPT1 phosphorylation on site 853, which was blocked by pretreatment with ROCK1 inhibitor Y-27632. COS-7 cells transfected with RhoA 88E/100E increased MYPT1 phosphorylation level compared to wild type RhoA and RhoA 88A/100A, suggesting the increased interaction of RhoA and ROCK1 enhanced ROCK1 activity (Fig 7D–7F).

### The effects of RhoA phosphorylation on its subcellular translocation

**Fig 8:**
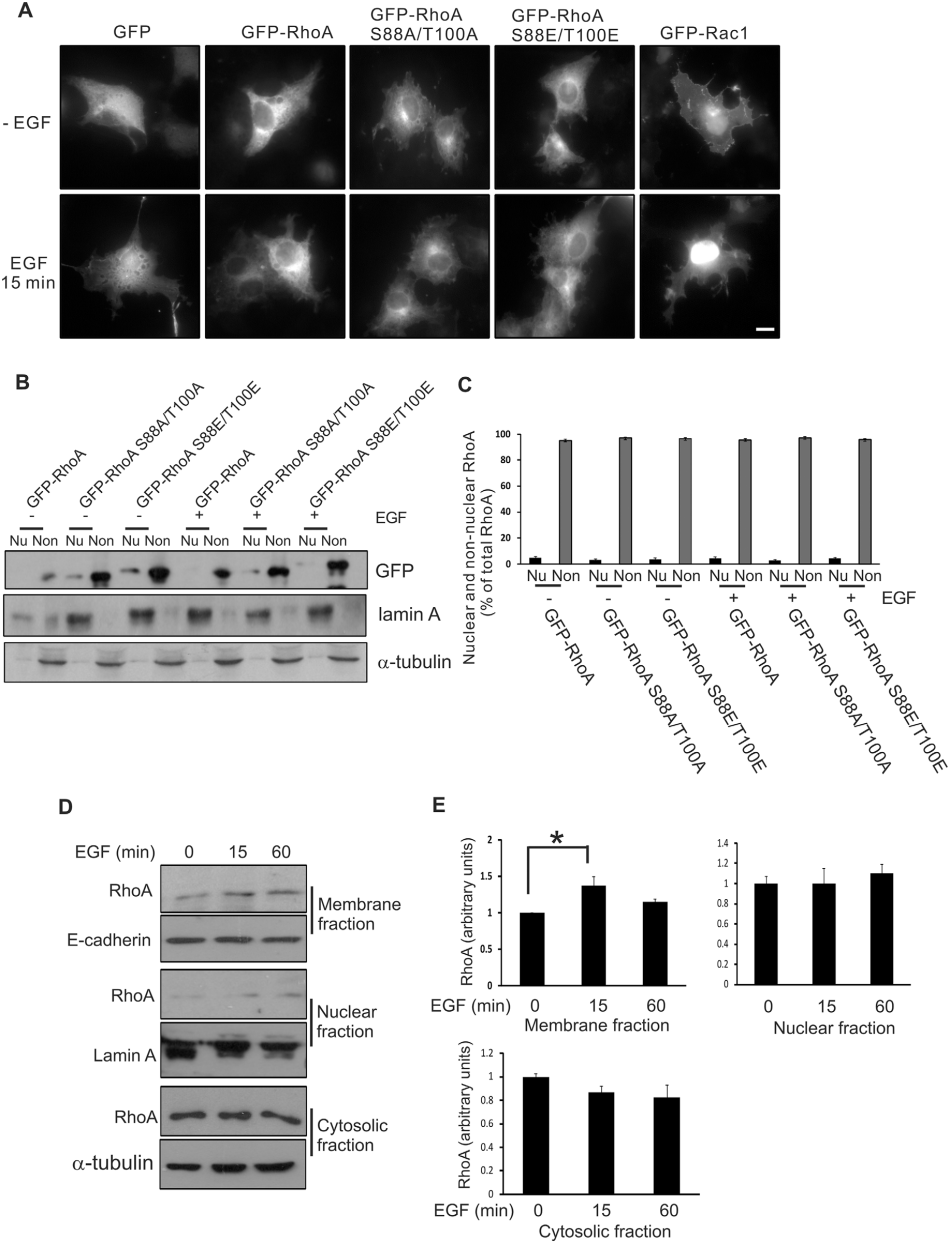
The effects of RhoA phosphorylation on its subcellular localization. **(A)** Subcellular localization of GFP-tagged wild type and mutant RhoA by fluorescence microscopy. COS-7 cells were transfected with expression constructs encoding GFP-tagged wild type or mutant RhoA proteins. Cells were either untreated or treated with EGF for 15 min. The localization of various RhoA proteins was observed by fluorescence microscopy. Size bar = 20 μm. **(B)** Subcellular localization of wild type and mutant RhoA by subcellular fractionation. COS-7 cells were transfected with expression constructs encoding GFP-tagged wild type or mutant RhoA proteins. The transfected COS-7 cells expressing GFP-proteins were homogenized, and the cell homogenates were separated into nuclear and non-nuclear fractions as described in Materials and Methods. The loading volumes of the nuclear fraction and non-nuclear fraction were about 25% and 3% of total sample volume, respectively, and were analyzed by SDS-PAGE and immunoblotting. Nu, nuclear fraction; Non, non-nuclear fraction. **(C)** Quantification of the data in (B). **(D)** Subcellular distribution of endogenous RhoA. After EGF stimulation for 15 and 60 min, lysates of COS-7 cells were separated into nuclear, total membrane, and cytosolic fractions as described in Materials and Methods. One-third of the nuclear fraction, one-half of the membrane fraction, and 3% of the cytosolic fraction were analyzed by immunoblotting. **(E)** Quantification of the data in (D). Each value is the mean of at least three experiments. The error bar is standard error. * indicates p<0.05.

Evidence is increasing that the subcellular localization of the Rho proteins plays a major role in their activation, and interaction with downstream effectors [7,40]. Although the majority of RhoA protein is localized in the cytosol and at the plasma membrane of cells, there have been reports that a fraction of the total RhoA pool is translocated to the nucleus and regulates downstream signaling [41–45]. We have shown previously that treatment of cells with EGF induced a significant amount of Rac1 to translocate from the cytosol into the nucleus [21]. In the present study, we examined whether the phosphorylation of RhoA would also induce its translocation into the nucleus. We first examined the subcellular localization of transiently-expressed GFP-tagged RhoA by fluorescence microscopy. As shown in Fig 8A, wild type RhoA or mutant RhoA S88A/T100A were mainly localized in the cytoplasm of untreated cells. EGF treatment failed to induce GFP-RhoA or GFP-RhoA S88A/T100A to translocate into the nucleus. Moreover, in contrast to the phosphomimetic GFP-Rac1 T108E that is mainly accumulated in the nucleus [21], phosphomimetic GFP-RhoA S88E/T100E was almost exclusively distributed in the cytoplasm with or without EGF stimulation (Fig 8A). These observations were confirmed by subcellular fractionation experiments (Fig 8B & 8C). COS-7 cells expressing GFP-RhoA and the mutants were either treated with EGF or not treated with EGF. Homogenates of these cells were fractionated into nuclear and non-nuclear fractions. As shown in Fig 8B & 8C, GFP-RhoA and the various mutants were mainly distributed in the non-nuclear fraction with or without EGF stimulation.

We also examined the effects of EGF stimulation on the subcellular localization of endogenous RhoA. As shown in Fig 8D & 8E, RhoA was mostly distributed in the cytoplasm without EGF stimulation. Following EGF stimulation for 15 min, a significant amount of RhoA was translocated to the plasma membrane, but not to the nucleus.

**Fig 9:**
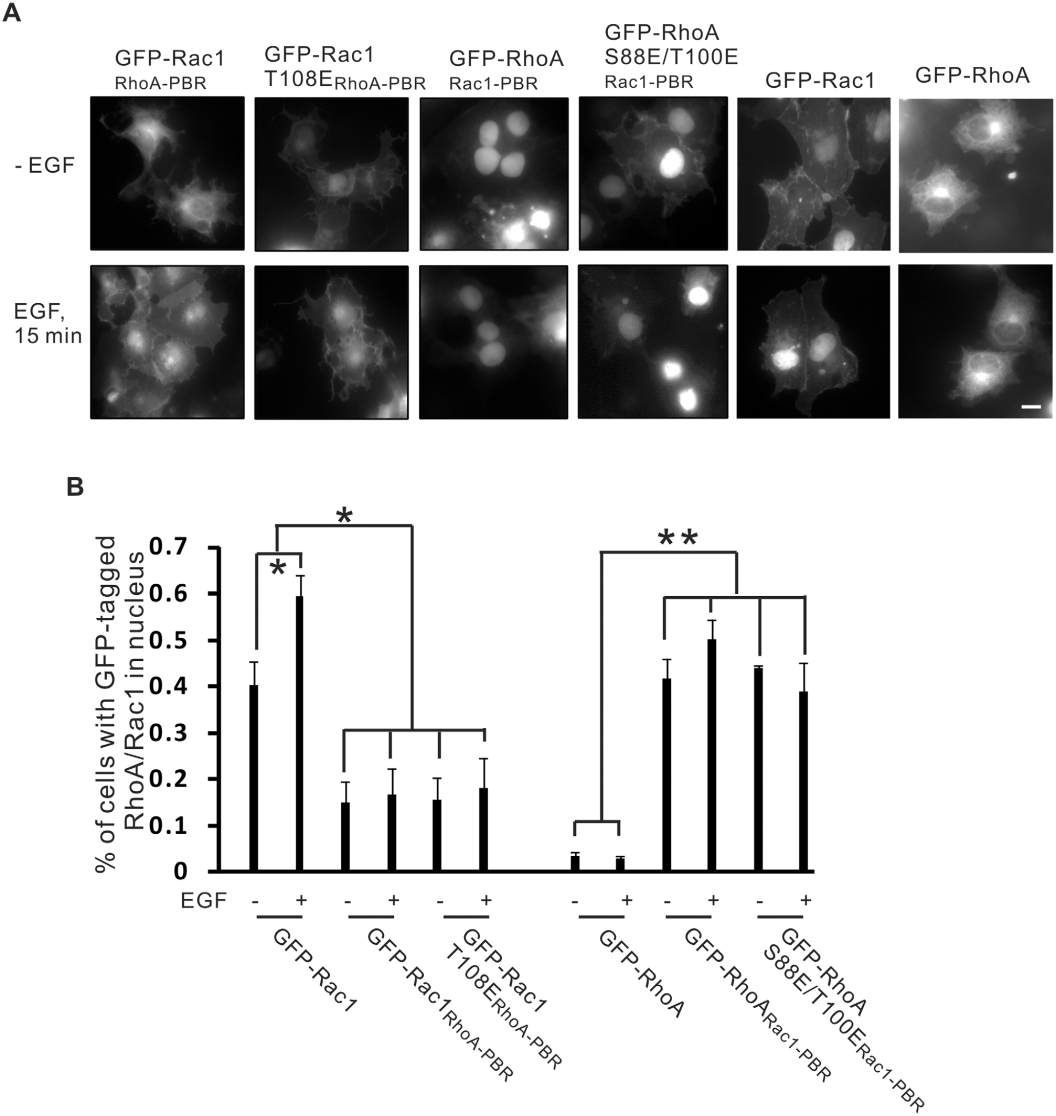
The effects of the polybasic region (PBR) and ERK-induced S/T phosphorylation on the nuclear localization of RhoA and Rac1. **(A)** COS-7 cells were transiently transfected with expression constructs encoding GFP-RhoA, GFP-Rac1, GFP-Rac1-T108E_RhoA-PBR_, GFP-RhoA-S88E/T100E_Rac1-PBR_, GFP-RhoA_Rac1-PBR_ and GFP-Rac1_RhoA-PBR_. Cells were either not treated or treated with EGF for 15 min. The localization of these wold type and mutant RhoA and Rac1 proteins was examined by fluorescence microscopy. Size bar = 20 μm. **(B)** Quantification of the data in (A). Each value is the mean of at least three experiments with at least 20 transfected cells counted for each experiment. The error bar is the standard error. *, p*<*0.05 and **, p<0.01.

It has been shown previously that Rac1 PBR (PVKKRKRK), containing a nuclear localization signal (NLS), promotes the accumulation of Rac1 in the nucleus, whereas RhoA PBR (RRGKKKSG), without an NLS, sequesters RhoA in the cytosol [42]. We have shown previously that the phosphorylation of Rac1 by ERK enhanced its nuclear translocation [21]. Here, we examined the roles of both the PBR and phosphorylation on the nuclear localization of RhoA and Rac1. We replaced the PBR of GFP-Rac1 T108E with RhoA PBR to generate the mutant GFP-Rac1 T108E_RhoA-PBR_. We also replaced the PBR of GFP-RhoA S88E/T100E with Rac1 PBR to generate GFP-RhoA S88E/T100E_Rac1-PBR_. These two mutants and two previously generated mutants (GFP-RhoA_Rac1-PBR_ and GFP-Rac1_RhoA-PBR_) were expressed in COS-7 cells by transient transfection. The localization of these four mutants was examined by fluorescence microscopy. As shown in Fig 9, Rac1 PBR targets RhoA to the nucleus with or without EGF stimulation and RhoA PBR targets Rac1 to the cytoplasm, which is consistent with a previous report [42]. Interestingly, RhoA PBR was able to target the phosphomimetic mutant Rac1 T108E to cytoplasm and Rac1 PBR was able to target the phosphomimetic mutants RhoA S88E/100E to the nucleus. These data suggest that PBR is the determining factor for the subcellular localization of RhoA and Rac1.

## Discussion

RhoA was the first Rho GTPase shown to be phosphorylated. It has been reported that RhoA S188 is phosphorylated by multiple kinases including PKA, PKG, AMPKα1and Mst3 kinase, and this phosphorylation inhibits RhoA activity and regulates multiple cellular processes, including the organization of actin cytoskeleton [6,11–16,36]. In the present study, we have shown that RhoA is phosphorylated at additional sites, S88 and T100, by activated ERK (Figs 1–3). We have reported previously that Rac1T108 is phosphorylated by activated ERK [21]. It is well documented that ERK phosphorylates the serine or threonine in the dipeptide motif S/T-P of target substrates, and there is some preference for proline at the -2 or -3 positions relative to the phosphorylated residue [27]. Moreover, the selection of the ERK substrate is dependent on ERK-docking sites (D-sites) with the core consensus motif (K/R)_1-3_-X_1-6_-φ-X- φ (where φ is a hydrophobic residue), located on ERK-interacting proteins [22,23]. Both RhoA and Rac1 contain the putative D-site at their C-termini, and these D-sites mediate the interaction between ERK and both RhoA and Rac1 (Fig 1, [21]. Although a P is missing at the -2 or -3 position of both S88 and T100, RhoA was more strongly phosphorylated by active ERK than Rac1. A possible explanation for this result is that RhoA contains multiple ERK phosphorylation sites including S88 and T100, but Rac1 only contains one site, T108. Indeed, mutation of both S88 and T100 simultaneously decreased RhoA phosphorylation much more than the mutation of either one of them (Fig 2). We also showed that Cdc42 is only marginally phosphorylated by active ERK (Fig 2). Although it has two PXTP motifs, Cdc42 does not have a strong D-site, which may explain its weak phosphorylation by ERK.

Our study is the first to demonstrate the direct interaction between ERK and RhoA, and that this interaction is dependent on the D-site of RhoA (Fig 1). We also showed that ERK can directly phosphorylate RhoA (Fig 2), RhoA phosphorylation is dependent on the activation of ERK in cells (Fig 3) and RhoA phosphorylation regulated RhoA activity (Fig 5). A body of evidence has shown that ERK and RhoA signaling pathways are closely linked. However, all of these studies examined the interaction between ERK and RhoA regulators, and the data are controversial. Some studies have shown that ERK enhances the activation of RhoA to regulate the actin assembly through phosphorylation of GEF-H1, a RhoA GDP/GTP exchange factor [46]. TNF-α-induced RhoA activation is mediated by ERK stimulation of GEF-H1 [47]. In contrast, another study has suggested that the phosphorylation of GEF-H1 by ERK inhibits GEF-H1 activity, which decreases RhoA activation [48]. This study further showed that the inhibition of ERK activity led to increased RhoA activation in certain Ras mutant cell lines [48]. Conversely, a different study has suggested that RhoA activation substantially prolongs the duration of ERK activation at both normal and reduced Ras levels [28]. Our present study provides a new mechanism for ERK to regulate RhoA.

RhoA phosphorylation by ERK on ^88^S and ^100^T increased RhoA activity (Fig 5), which is opposite to the effects of RhoA S188 phosphorylation, as reported previously (5, 6, 11). RhoA has been reported as a target for several protein kinases. PKAPKG are two main kinases that phosphorylate RhoA on serine 188 [6,11–14]. RhoA is also phosphorylated by AMPKα1 Mst3 kinase [15,16]. RhoA phosphorylation on serine 188 by PKA and PKG did not modify its GTPase activity and its interaction with GEFs and GAPs; however, it deactivates RhoA by increasing its interaction with RhoGDI and translocation of RhoA from the membrane to the cytosol [5,6,11,12]. Our data indicate that RhoA phosphorylation at different sites regulates RhoA differently.

The effects of ERK-induced RhoA phosphorylation on RhoA activity is also opposite to the effects of ERK-induced Rac1 phosphorylation on Rac1 activity. We have shown previously that phosphorylation of Rac1 by ERK decreased Rac1 activity [21]. It is well documented that co-ordinated regulation of RhoA and Rac1 activity is important for many cellular functions. For example, Rac1 and RhoA have been shown to exhibit mutual antagonism in migrating cells [49–51]. Mutual antagonism also produces balanced activities of RhoA-generated apical constriction and Rac1-dependent cell elongation that control cell shape, and thus, the curvature of the invaginating epithelium [52]. We propose that by phosphorylating both RhoA and Rac1, ERK is able to increase RhoA activity and decrease Rac1 activity. Thus, ERK is well positioned to regulate the cell functions that require the mutual antagonism of RhoA and Rac1.

In the present study, we have further shown that ERK-induced RhoA phosphorylation enhances the formation of actin stress fibers (Fig 6), which is consistent with the increased RhoA activity induced by ERK-mediated RhoA phosphorylation. It is interesting to note that RhoA phosphorylation on serine 188 induces the collapse of actin stress fibers [6,13]. To gain insight into the mechanisms underlying the regulation of actin stress fiber formation by ERK-induced RhoA phosphorylation, we examined the effects of RhoA phosphorylation on its interaction with downstream substrates. Several RhoA substrates, including ROCK1 and mDia, have been implicated in mediating the RhoA regulation of actin remodeling [33,35]. Here, we have shown that RhoA phosphorylation enhances its interaction with ROCK1, but does not affect its interaction with mDia (Fig 7). We further showed that RhoA phosphorylation on ^88^S and ^100^T increase ROCK1 activity and the phosphorylation of ROCK1 substrate NYPT1 (Fig 7). Thus, it is likely that ROCK1 is responsible for the enhanced actin stress fiber formation induced by RhoA phosphorylation on ^88^S and ^100^T. Consistent with our results, it has been found that the phosphorylation of serine 188 on RhoA by PKA induced by nerve growth factor (NGF) blocks RhoA association with ROCK1 without affecting its ability to interact with other effectors including rhotekin, mDia, and PKN [53].

It has been reported that in PANC-1 cells, EGF treatment induces RhoA translocation from the cytosol to the membrane fraction, and actin stress fiber assembly [54]. However, using a fluorescence resonance energy transfer based RhoA probe assay, the activity of RhoA was greatly decreased at the plasma membrane in EGF-stimulated COS-1 and NIH3T3 cells [55]. Our present study showed that EGF stimulated the translocation of RhoA to the plasma membrane and enhanced actin stress fiber formation. We also showed that actin stress fiber formation was enhanced in cells transfected with phosphomimetic mutant RhoA S88E/T100E. These data indicate that EGF has a positive effect on RhoA activation and stress fiber formation that is probably mediated by ERK.

It is now well established that the subcellular location of the active Rho protein plays an important role in how it becomes activated and the downstream effectors with which it interacts. Rho proteins are synthesized as inactive cytosolic proteins and are targeted to the plasma membrane upon specific activation by GEFs or by virtue of a series of posttranslational modifications of the C-terminal CAAX motif [56]. However, the dogma that active Rho proteins are localized to the plasma membrane while inactive Rho proteins are in the cytosol is overly simplistic. The subcellular localization of Rho proteins is more complex than initially proposed [40,56]. Both Rac1 and RhoA have been found to be localized to the nucleus [21,57–59]. Although the majority of RhoA is localized in the cytosol and at the plasma membrane of cells, there are reports that a fraction of the total RhoA pool is distributed to the nucleus and regulates downstream signaling [41,43–45,60].

Like other members in the Rho proteins family, both Rac1 and RhoA have a polybasic region (PBR) in their C-termini that consists of multiple basic lysines and arginines, which is adjacent to and immediately precedes the C-terminal CAAX sequence. Besides the prenylation of the CAAX motif and the interaction with RhoGDI, the subcellular localization of Rac1and RhoA is also regulated by its PBR. It has been shown that the Rac1 PBR (PVKKRKRK) contains a nuclear localization signal (NLS) and thus promotes Rac1 nuclear accumulation, whereas the RhoA PBR (RRGKKKSG) lacks a NLS and sequesters RhoA in the cytosol [42].

Since we have shown that Rac1 phosphorylation on T108 by ERK plays important role in targeting Rac1 to the nucleus [21], in the present study we examined whether ERK-induced RhoA phosphorylation is important in determining the subcellular localization of RhoA. We showed that treatment with EGF for 15 min, which activates ERK and induces RhoA phosphorylation, increased the plasma membrane localization of endogenous RhoA, but had no effects on the nuclear localization of RhoA (Fig 8A & 8B). We also showed that the phosphomimetic mutant GFP-RhoA S88E/T100E has a similar level of nuclear localization with or without EGF stimulation, as does the non-phosphorylated mutant GFP-RhoA S88A/T100A (Fig 8C–8E). These data indicate that RhoA phosphorylation by ERK does not target RhoA to the nucleus, in contrast to Rac1 phosphorylation by ERK.

We also examined the relative importance of the PBR and ERK-induced phosphorylation on the subcellular localization of RhoA and Rac1. Our data indicate that the PBR is the determining factor for the subcellular localization of both RhoA and Rac1. Rac1 PBR domain is able to target significant amount of RhoA to the nucleus regardless the phosphorylation status of RhoA (Fig 9). On the other hand, RhoA PBR is able to significantly reduce the nuclear localization of Rac1 regardless of the phosphorylation status of Rac1 (Fig 9). EGF-induced nuclear translocation of Rac1 is dependent on ERK-induced Rac1 T108 phosphorylation, as we have reportedly previously [21]. However, EGF-induced nuclear translocation of Rac1 also requires the presence of Rac1 PBR, as EGF fails to stimulate the nuclear translocation of GFP-Rac1_RhoA-PBR_ (Fig 9). It has been shown that switching of the PBRs between Rac1 and RhoA alters their nuclear accumulation [42].

ERK plays a pivotal role in the mitogenic signal transduction pathway. ERK cascades are critical in regulating cell proliferation, survival and differentiation. The aberrant regulation of ERK cascades contributes to cancer and other human diseases [61]. Constitutive activation of ERK has been observed in many tumor cell lines in a tissue-specific manner [62]. Like many other members in Rho GTPases family, increased RhoA expression has often been correlated with human cancer progression through its regulation of cell migration and is linked to poor prognosis [63–67]. Moreover, RhoA is both overexpressed and spontaneously active in the invasive breast cancer cell line MDA-MB-231 [68]. The ectopic expression of RhoA can induce the paraneoplastic transformation of mammary epithelial cells [69]. In our present study, it is noteworthy that a direct physical association between RhoA and ERK can be found both in the COS-7 cell line and in SKBR3, a breast cell line. In addition, this association is stronger in SKBR3 cells than in COS-7 cells (Fig 1C & 1D). The physiological significance of this interaction requires further research.
